# Metabolite profiling and adaptation mechanisms of *Aspergillus cristatus* under pH stress

**DOI:** 10.3389/fmicb.2025.1576132

**Published:** 2025-04-01

**Authors:** Rongrong Zhang, Lihong Zhou, Luyi Xie, Lingqing Lu, Hang Zhou, Yi Yang, Jiuping Hu

**Affiliations:** ^1^Key Laboratory of Plant Resource Conservation and Germplasm Innovation in Mountainous Region (Ministry of Education), College of Life Sciences, Institute of Agro-Bioengineering, Guizhou University, Guiyang, China; ^2^PingBa No.1 Senior High School in Anshun City, Anshun, China; ^3^Ya’an Xunkang Pharmaceutical Co., Ltd., Ya’an, China

**Keywords:** *Aspergillus cristatus*, pH stress, different substances, pH homeostasis, adaptation mechanism

## Abstract

**Introduction:**

pH is an important environmental factor affecting the survival of fungi, and *Aspergillus cristatus*, which can grow and reproduce over a wide range of pH, is suitable for studying their adaptation mechanism to pH stress.

**Methods:**

In this study, *A. cristatus* was cultured on plates of different initial pH (pH 3.8-8.0), with the results revealing distinct morphologies at pH 3.0–5.0, pH 6.0–7.0 and pH 8.0. Liquid chromatography-mass spectrometry (LC-MS) and multivariate analysis subsequently were used to analyze the changes of substance metabolism of *A. cristatus* at different pH.

**Results and discussion:**

LC-MS and multivariate analyses showed that *A. cristatus*’s growth at different pH involved significantly different metabolites. In particular, comparing pH 4.0 vs pH 6.0, pH 6.0 vs pH 8.0 and pH 4.0 vs pH 8.0 revealed a total of 317, 171 and 404 significantly different substances, respectively. Finally, as the pH changed from 4.0 to 6.0 to 8.0, eight changes in the patterns of differential substances were identified. At low pH, *A. cristatus* accumulated large amounts of energy substances (e.g., adenosine), active antioxidants (e.g., glutathione) and osmo-protective substances (e.g., raffinose). In contrast, at high pH, large amounts of phosphatidylcholine (PC), lysophosphatidyl ethanolamine (LPE), lysophosphatidyl choline (LPC), lysophosphatidyl serine (LPS) related to biofilms were synthesized, alongside antioxidants (e.g., formononetin) and acidic substances. The aforementioned results indicate that *A. cristatus* adapts to changes in pH by adjusting their metabolite synthesis. Therefore, under unsuitable pH environments, *A. cristatus* synthesizes specific sets of metabolites that play key roles to cope with the stress.

## 1 Introduction

Fungal growth occurs over a wide range of pH, and this explains their distribution in environments with highly different pH. For instance, *Aspergillus niger* (Schuster et al., 2002), *Aspergillus fumigatus* (Toledo et al., 2022), *Aspergillus nidulans* (Caddick et al., 1986) and *Candida albicans* (Davis, 2003) can survive within a pH range of 1.4–9.8, 2.1–8.8, 3.5–9 and 2.0–10.0, respectively. Although these fungi exhibit remarkable adaptability to a wide range of pH levels, their optimal growth occurs within a much narrower range. Nevertheless, fungi are capable of surviving beyond their optimal growth pH, thus highlighting their ability to respond to long-term environmental changes.

How do fungi respond to unfavorable pH conditions? According to existing research, they cope with pH stress through two main strategies: by maintaining the homeostasis of the intracellular pH (pH_i_) (Palmgren, 2023) and by regulating the production of extracellular enzymes and permeases through the Pal/Rim genetic regulatory system (Cornet and Gaillardin, 2014; Trushina et al., 2013). The maintenance of pHi homeostasis represents a fundamental physiological strategy in fungi, essential for preserving enzymatic functionality, stabilizing macromolecular conformations, and ensuring proper biomolecular interactions. This critical regulatory mechanism involves the plasma membrane-embedded P-type ATPase PAM1 and vacuolar-type ATPase (V-ATPase) complexes, which work together with other transporters to maintain pH_i_ homeostasis (Kane, 2016; Young et al., 2024). For example, under acidic stress, yeast can activate PAM1 and V-ATPase in the presence of glucose to adjust the intracellular pH to a more neutral or lightly alkaline level (Serrano, 1983). In addition, fungi possess a conserved pH-dependent genetic regulatory system: the Pal system in filamentous fungi and the Rim system in yeast (Caddick et al., 1986; Peñalva et al., 2008; Denison, 2000). In response to changes in environmental pH, fungi can activate the specific transcription factor PacC/Rim101 via the Pal/Rim signaling pathway, and in doing so, they can regulate the synthesis of extracellular enzymes (e.g., acid phosphatase, alkaline phosphatase and phosphodiesterase) and permeases (Pianalto et al., 2024; Caddick et al., 1986; Selvig and Alspaugh, 2011). By regulating the expression of genes involved in the synthesis of these molecules, fungi ensure that they are produced only at pH levels where they can effectively function. For example, *A. nidulans* (Caddick et al., 1986) synthesizes acid phosphatase under acidic conditions and alkaline phosphatase in alkaline conditions. This genetic regulation represents the second mechanism through which fungi adapt to varying pH environments.

Besides the two previously mentioned mechanisms, fungi can also secrete organic acids or alkaline substances into their external environment to regulate pH. For instance, *A. niger* (Andersen et al., 2009; Poulsen et al., 2012) produces large amounts of citric acid, oxalic acid and gluconic acid to regulate environmental pH, while *Aspergillus carbonarius* (Barda et al., 2020) secretes gluconic acid and citric acid to acidify the environment under alkaline conditions. Similarly, *Aspergillus oryzae* (Akasaka et al., 2018) was shown to increase the production of agmatine in response to acidic stimulation. Fungi can also respond to oxidative stress and cellular damage caused by varying pH levels by regulating the synthesis of antioxidants and osmolytes. For example, a study by Li et al. (2019) found that low pH stress in *Pichia kudriavzevii* cells enhanced the level of glutathione and the activity of antioxidant enzymes. Furthermore, the alkaliphilic fungus *Sodiomyces tronii* accumulated trehalose as an osmotic regulator at pH 5.4, while mannitol and arabitol were more prevalent at pH 10.2 (Bondarenko et al., 2017). Finally, some fungi, such as *Yarrowia lipolytica* (González-López et al., 2006), *Penicillium marneffei* (Cao et al., 2007) and *Candida albicans* (Buffo et al., 1984), can adapt to different pH environments by altering their vegetative growth mode, resulting in their growth mainly as yeast in acidic environments and as filaments in alkaline ones. Overall, fungi employ a wide range of strategies to adapt to different environmental pH, with different species exhibiting distinct methods of adaptation.

*Aspergillus cristatus*, an important edible fungus, can grow in a pH range of 3 to 9, and as such, it represents an ideal test organism for studying the mechanism of pH adaptation. So far, research on the effects of pH on *A. cristatus* has largely focused on changes in morphology and pigmentation. For instance, Liu and Qin (1991) and Zheng (2015) reported variations in the colony diameter, colony color, yellow pigment and sporulation mode of *A. cristatus* at different environmental pH. Therefore, the results of existing studies have been mostly limited to descriptions of phenotypic changes, without an in-depth study of the underlying mechanism. In contrast to previous research, this work investigates the changes that occur at the metabolomics level when *A. cristatus* grows at different initial pH, including optimum and non-optimum ones, as well as the adaptation mechanisms of *A. cristatus* to pH stress was discussed from the perspective of metabolites. It is expected that the findings will provide a basis for understanding the potential mechanism through which *A. cristatus* responds to unfavorable pH environments.

## 2 Materials and methods

### 2.1 Strains

*A. cristatus* CGMCC 3.17718 was purchased from the China General Microbiological Culture Collection and Management Center (Beijing, China).

### 2.2 Medium

Potato dextrose agar (PDA) (Mislivec and Bruce, 1976) was used for strain preservation and establishing slant seed culture, while Czapek’s agar medium (CZA) (Xie et al., 2024) was used for culturing *A. cristatus* and the preparation of mycelium samples.

### 2.3 Reagents

All reagents, namely ethanol (≥ 99.8%, Thermo Fisher, United States), methanol (≥99.0%, Thermo Fisher, United States), formic acid (LC–MS Grade, Thermo Fisher, United States), ammonium formate (LC–MS Grade, Thermo Fisher, United States), H_2_O (LC–MS Grade, Merck, Germany), hydrochloric acid (≥ 99%, Sinopharm Chemical Reagent Co., Ltd., China) and sodium hydroxide (≥ 96.0%, Chengdu Jinshan Chemical Reagent Co., Ltd., China) were analytically pure.

### 2.4 Instruments and equipment

Vertical pressure steam sterilization pot (LDZX-50 KBS, Shanghai, China), mold incubator (TAISITE, MJX-series, Tianjin, China), electronic balance (FA1004N, Shanghai, China), pH meter (INESA, PHS-25, Shanghai, China), light microscope (Olympus, BX53F, Tokyo, Japan), microcentrifuge (Beckman coulter, Allegra X-30R, Germany), vortex mixer (Kylin-bell, BE-2600, Haimen, China), constant-temperature water bath with a digital display (MX15H135-A12E, Niles, IL, United States), low-temperature centrifuge (Scilogex, D3024R, United States), liquid chromatograph (Thermo, Vanquish, Germany), chromatographic column (Thermo, Hypersil Gold column, United States) and mass spectrometer (Thermo, Q Exactive™ HF, Germany) were used in this study.

### 2.5 Culture of *Aspergillus cristatus* on media of different initial pH

#### 2.5.1 Media preparation

The CZA culture medium was prepared according to the provided instructions before being sterilized for 20 min at 121°C and a pressure of ≤0.24 Mpa. After cooling to 50–60°C, the pH value was adjusted to 4.0 ± 0.2, 6.0 ± 0.2 or 8.0 ± 0.2 with 1.0 mol/L HCl or 1.0 mol/L NaOH solution, as required. The initial pH of all media was confirmed with a pH meter (INESA, PHS-25, Shanghai, China).

#### 2.5.2 Culture of *Aspergillus cristatus* and preparation method of mycelium

*A. cristatus* strains that had been cultured for 7 days at 25 ± 0.5°C were inoculated onto CZA plates of different initial pH. The plates were then incubated for 12 days at a constant temperature of 25 ± 0.5°C and a relative humidity of 60–80% before being observed for morphological changes. In addition, the mycelia were collected in sterile EP tubes and stored at −80°C for LC–MS non-targeted metabolomics analysis. For each pH, morphological observation and mycelial collection were performed for six replicates.

### 2.6 Morphological observation of *Aspergillus cristatus* at different initial pH

The morphological characteristics of *A. cristatus*’s colonies were observed and described. In particular, details on the colony’s color, texture, shape and other morphological characteristics were recorded. Furthermore, morphological features, as observed under a microscope, were visualized alongside signs of sporulation using the sticking method. This method involved sticking a piece of transparent tape on the colony mycelium along the direction of colony growth. The tape was then placed on a cover glass containing a drop of cotton blue dye, with the cover glass subsequently placed on a microscope slide for observation.

### 2.7 Measurement of colony diameter, biomass and number of asexual spores

The colony diameter of cultured *A. cristatus* was determined using the cross method. The whole mycelium was then collected and dried to a constant weight in an oven at 90 ± 1°C. In addition, three pieces of mycelium, taken from the center of the colony with a puncher of pore size 0.7 cm, were placed in a 5 mL EP tube. Tween 80 solution (1 mL, 0.05%) as well as an appropriate amount of glass beads were subsequently added before mixing for 1 h on a rapid mixer. The mixture was filtered through four layers of wiped mirror paper, and the number of asexual spores in the resulting filtrate was eventually counted using a blood cell counting plate. The above experiment was repeated six times, and the number of asexual spores was considered as 0 if it was less than 1 × 10^4^ cfu/cm^2^.

### 2.8 LC–MS non-targeted metabolomics analysis

#### 2.8.1 Sample preparation

Mycelium samples of cultured *A. cristatus* were accurately weighed (60 mg) and transferred to EP tubes where they were suspended in prechilled 80% methanol (300 μL). After vortex mixing, the samples were frozen in liquid nitrogen for 5 min. After which they were allowed to melt on ice before being vortexed for 30 s. The samples were then sonicated for 6 min prior to a 1 min centrifugation at 5,000 rpm and 4°C. The resulting supernatant was freeze-dried and after being dissolved in 300 μL of 10% methanol, it was filtered through a 0.22 μm membrane before being eventually injected into the LC–MS/MS system.

Equal volumes of samples were collected from each test sample and mixed to obtain the QC samples. These QCs were then used to test the instruments, equilibrate the UHPLC–MS/MS system before sample injection and examine the stability of the system during the analytical procedure.

#### 2.8.2 UHPLC–MS/MS analysis

UHPLC–MS/MS was performed using a Vanquish UHPLC system (Thermo Fisher, Germany), coupled with an Orbitrap Q Exactive™ HF mass spectrometer or Orbitrap Q Exactive™ HF-X mass spectrometer (Thermo Fisher, Germany). The samples were first injected into a Hypersil Gold column (100 × 2.1 mm, 1.9 μm), with the eluents for the positive polarity mode being eluent A (0.1% formic acid in water) and eluent B (methanol), while those of the negative polarity mode were eluent A (5 mM ammonium acetate, pH 9.0) and eluent B (methanol). Separation was achieved using a flow rate of 0.2 mL/min and the following 17 min linear gradient: 2% B, 1.5 min; 2–85% B, 3 min; 85–100% B, 10 min; 100–2% B, 10.1 min; 2% B, 12 min.

Furthermore, for the Q Exactive™ HF mass spectrometer, operated in the positive/negative polarity modes, the following parameters were applied: a spray voltage of 3.5 kV, a capillary temperature of 320°C, a sheath gas flow rate of 35 arb, an aux gas flow rate of 10 arb, an S-lens RF level of 60 and an aux gas heater temperature of 350°C.

#### 2.8.3 Data preprocessing and metabolite identification

The raw data files generated by UHPLC–MS/MS were processed using Compound Discoverer 3.3 (CD3.3, Thermo Fisher) to perform peak alignment, peak picking as well as quantification of each metabolite. For this purpose, the following main parameters were set: an actual mass tolerance of 5 ppm, a signal intensity tolerance of 30%, and a minimum intensity, et al., while the peak area was corrected using the first QC. The peak intensities were subsequently normalized to the total spectral intensity before being used to predict the molecular formula based on additive ions, molecular ion peaks and fragment ions. The peaks were also matched against the mzCloud^1^, mzVault and Mass List databases to obtain accurate qualitative and relative quantitative results. The results were then statistically analyzed using the statistical software R (R version R-3.4.3), Python (Python 2.7.6 version) and CentOS (CentOS release 6.6). In the case of non-normally distributed data, standardization was first performed according to the following formula: sample raw quantitation value/(sum of sample metabolite quantitation value/sum of QC1 sample metabolite quantitation value) to obtain relative peak areas. And compounds whose coefficient of variation of relative peak areas in QC samples were greater than 30% were removed, and finally the metabolites’ identification and relative quantification results were obtained.

These metabolites were annotated using the KEGG database^2^, HMDB database^3^ and LIPIDMaps database.^4^

### 2.9 Gamma-glutamylcysteine synthetase enzyme activity validation

The γ-GCS enzyme activity was assessed using the γ-GCS Enzyme Activity Assay Kit of Suzhou Gris Biotechnology Co., LTD (G0214F, Jiangsu, China).

### 2.10 Statistical and multivariate analyses

The metabolic profiles from both positive and negative modes were used for subsequent analysis and classification. The data were first imported into SIMCA-P software (Umetrics AB, Umea, Sweden) for principal component analysis (PCA), orthogonal partial least squares discriminant analysis (OPLS-DA) and other multivariate statistical analyses in order to identify the metabolic changes induced in *A. cristatus* under different pH conditions. Specifically, PCA was used to fit the overall data, detect internal patterns within the data matrix and distinguish differences between samples of *A. cristatus* that had been exposed to different pH conditions. OPLS-DA was used to classify the samples based solely on Y variables. In addition, volcano maps, Venn diagrams and box plots were generated using the microbiome cloud platform.^5^ By calculating the fold change (FC) of metabolites, we could determine whether the metabolites have changed between the groups, the extent of change and whether the difference is significant. Combined with the *p*-value, some crucial metabolites can be screened out, and then combined with the difference in the weight contribution value (VIP) (The contribution of metabolites with VIP > 1 to the discriminant analysis was larger, and these metabolites were significantly different between the groups), substances contributing significantly to the differentiation of varying samples were screened. Furthermore, the topological analysis and enrichment analysis were combined to screen for key metabolic pathways. Firstly, metabolites with *p* < 0.05 were screened through a *t*-test, and then, the metabolic pathways in which these metabolites appeared were identified. By calculating the Over Representation Analysis *p*-values of these metabolic pathways, we identified the significant enrichment pathways of differential metabolites. Finally, topological analysis was performed to calculate the magnitude of the effect of metabolites in the metabolic pathways (measured by impact). The identified significant differential metabolites were treated as X variables, while the biomass and colony diameter of *A. cristatus* were used as Y variables for correlation analyses. The correlation index was calculated by Pearson’s correlation method using the OmicShare tools, a free online platform for data analysis (http://www.omicshare.com/tools, accessed on 29 Aug 2024). All statistical analyses were performed with SPSS Statistics 26 software, with GraphPad 9.5.1 used for data visualization. The data are presented as mean ± standard error.

## 3 Results

### 3.1 Characteristic morphology of *Aspergillus cristatus* at different initial pH

*A. cristatus* was cultured on CZA plates of different initial pH for 12 days at a constant temperature and relative humidity. Overall, the colonies exhibited distinct characteristics, including at microscopic levels, at pH ranges 3.0–5.0, 6.0–7.0 and 8.0 (Supplementary Figures S1, S2).

**Figure 1:**
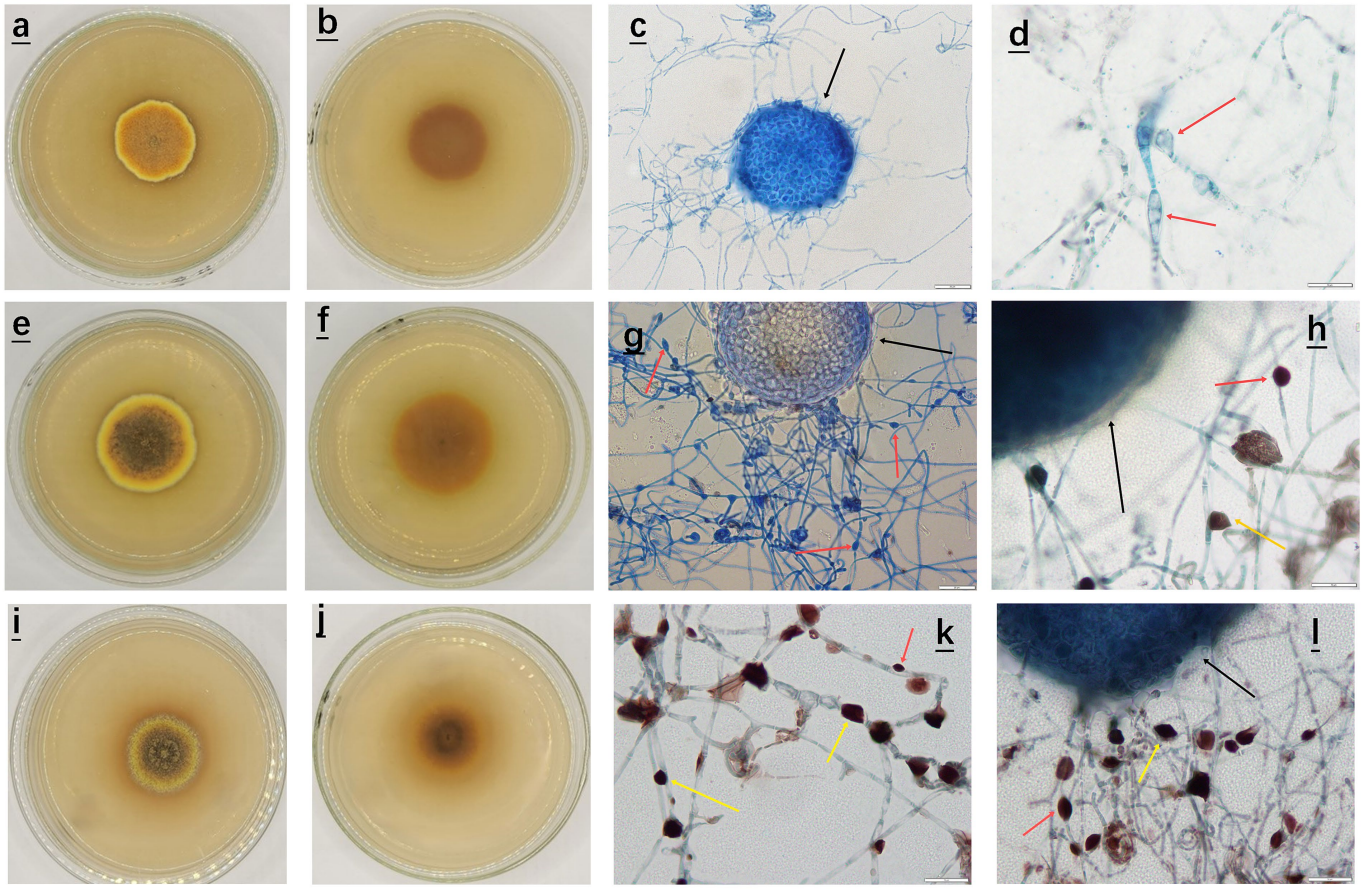
Morphological characteristics of *A. cristatus* at different pH. **(a–d)** Front, back and sporulation structure of the colony at pH 4.0; **(e–h)** Front, back and sporulation structure of the colony at pH 6.0; **(i–l)** Front, back and sporulation structure of the colony at pH 8.0. **(c,g)** Sporulation structure and spore morphology of *A. cristatus* as observed under a microscope at a magnification of 40x, scale: 20 μm; **(d,h,k,l)** Sporulation structure and spore morphology of *A. cristatus* as observed under a microscope at a magnification of 100x, scale: 10 μm.

Specifically, in the pH range of 3.0–5.0, *A. cristatus* had nearly round, thin and flat colonies, with the texture being dense and felt-like. The morphological characteristics results, shown for pH 4.0, are presented in Figures 1a–d. Furthermore, the front and back of the colony were orange red and yellowish brown, respectively, while the outermost circle was yellow. Finally, cleistothecium formed by sexual reproduction, was visible alone or in strings under an optical microscope (black arrow in Figure 1), with very few nearly elliptical spores (red arrow in Figure 1) also observed.

In the pH range of 6.0 to 7.0, *A. cristatus* had nearly round colonies but they were thicker and had a denser texture compared with those observed at pH 4.0. These morphological observations results are presented for pH 6.0 in Figures 1e–h. The surface of the outer ring mycelium was also villous, while its middle part was entangled into small floccules. In terms of color, the front of the colony gradually changed from dark brown to yellowish brown from the center to the periphery. In addition, the outermost circle was yellow, while the back of the colony was dark brown to yellowish brown. The cleistothecium (alone or in strings), formed by sexual reproduction, was also observed under an optical microscope (black arrow in Figure 1), alongside a large number of nearly oval (red arrow in Figure 1) or irregular (yellow arrow in Figure 1) spores formed by asexual reproduction.

Finally, at pH 8.0, *A. cristatus* exhibited nearly round colonies (Figures 1i–l) having a dense texture and a thickness between that observed for pH 4.0 and pH 6.0. The surface also consisted of short hyphae entangled into small floccules, with the central hyphae being slightly uplifted and hard. The colony also formed a concentric ring of yellow and dark brown alternating from the center to the periphery. In addition, the cleistothecium (alone or in strings), formed by sexual reproduction, could be observed under an optical microscope (black arrow in Figure 1), alongside a large number of nearly oval (red arrow in Figure 1) or irregular (yellow arrow in Figure 1) spores formed by asexual reproduction.

**Figure 2:**
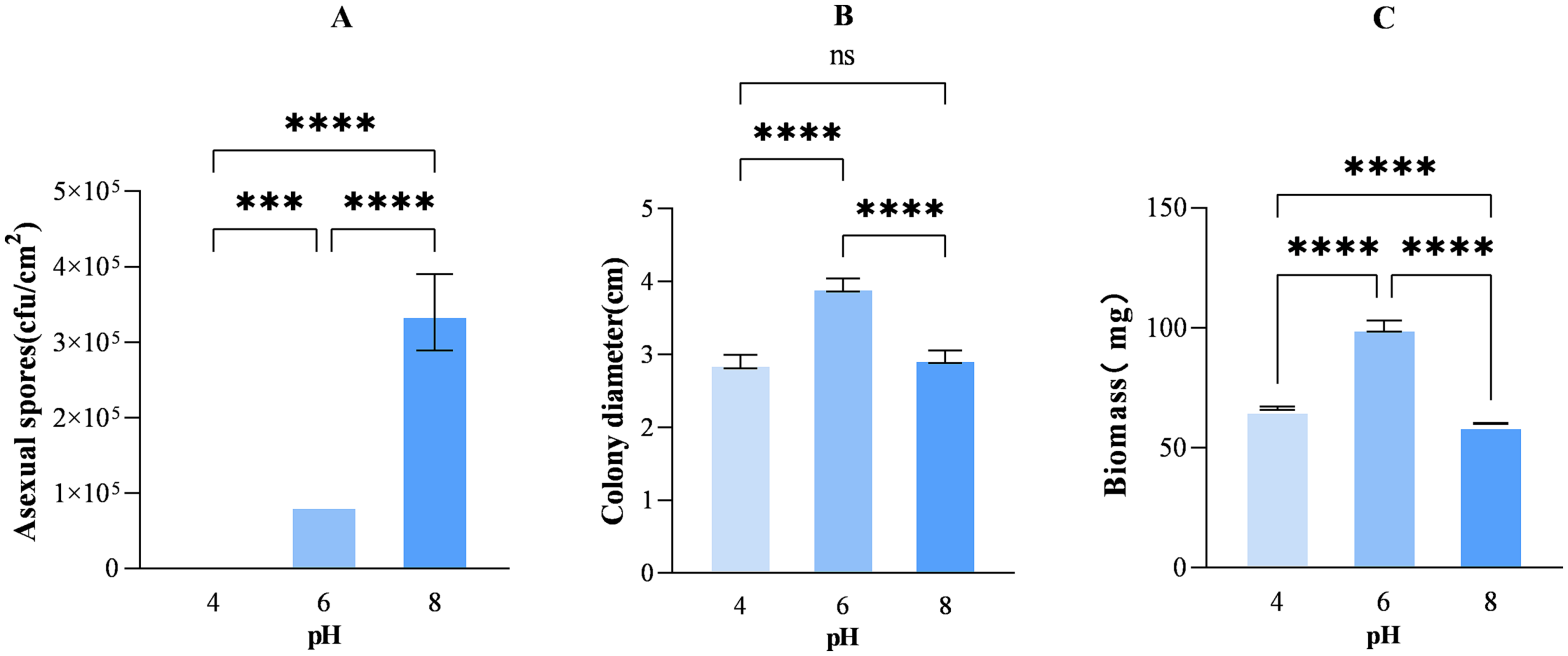
The number of asexual spores per cm^2^ **(A)**, colony diameter **(B)** and biomass **(C)** of *A. cristatus* after 12 days of growth on media of different pH. *** Means *p* < 0.001, ****means *p* < 0.0001, ns means *p* > 0.05.

In addition, the number of asexual spores produced by *A. cristatus* at pH 4.0, 6.0 and 8.0 after 12 days of growth was determined, and the results showed significant differences in the number of spores produced at the three different pH (Figure 2A). Specifically, the lowest and highest numbers were obtained at pH 4.0 (less than 1 × 10^4^ cfu/cm^2^) and pH 8.0 ((3.39 ± 0.51) × 10^5^ cfu/cm^2^), respectively, while that obtained at pH 6.0 was between the two at 8.67 × 10^4^ cfu/cm^2^. Altogether, the findings indicate that *A. cristatus*’s ability to form asexual spores increases at higher environmental pH, thus presenting the pH as a crucial factor that regulates asexual reproduction in this particular fungus.

To explore the vegetative growth of *A. cristatus* in different initial pH environments, the colony diameter and biomass were further determined. The results displayed that the colony diameters of *A. cristatus* at pH 4.0, 6.0 and 8.0 were about 2.9 ± 0.09 cm, 3.95 ± 0.08 cm and 2.97 ± 0.08 cm, respectively. The colony diameter at pH 6.0 was significantly larger than that at pH 4.0 and pH 8.0, and there was no significant difference between the latter two (Figure 2B). In addition, the biomass of *A. cristatus* at pH 6.0 was 100.73 ± 2.25 mg, which was significantly higher than that at pH 4.0 and pH 8.0, which were 66.50 ± 0.74 mg and 60.12 ± 0.23 mg, respectively (Figure 2C). These results demonstrate that it is beneficial to the vegetative growth of *A. cristatus* at the pH 6.0. It is also suggested that the environmental pH deviating from the pH 6.0 will inhibit the vegetative growth of *A. cristatus*, and it can also be considered as the growth under pH stress that deviates from the optimal pH range.

### 3.2 The metabolic profiles of *Aspergillus cristatus* at different initial pH

**Figure 3:**
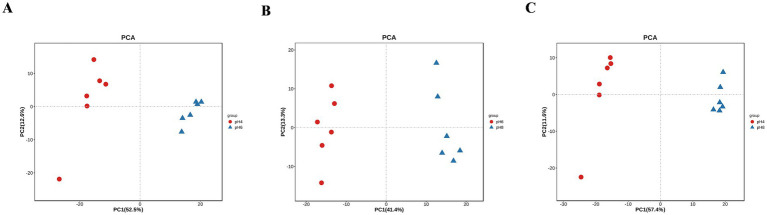
PCA of samples obtained at pH 4.0, pH 6.0 and pH 8.0. **(A–C)** respectively represent the PCA analysis scores of pH 4.0 vs. pH 6.0, pH 6.0 vs. pH 8.0 and pH 4.0 vs. pH 8.0.

**Figure 4:**
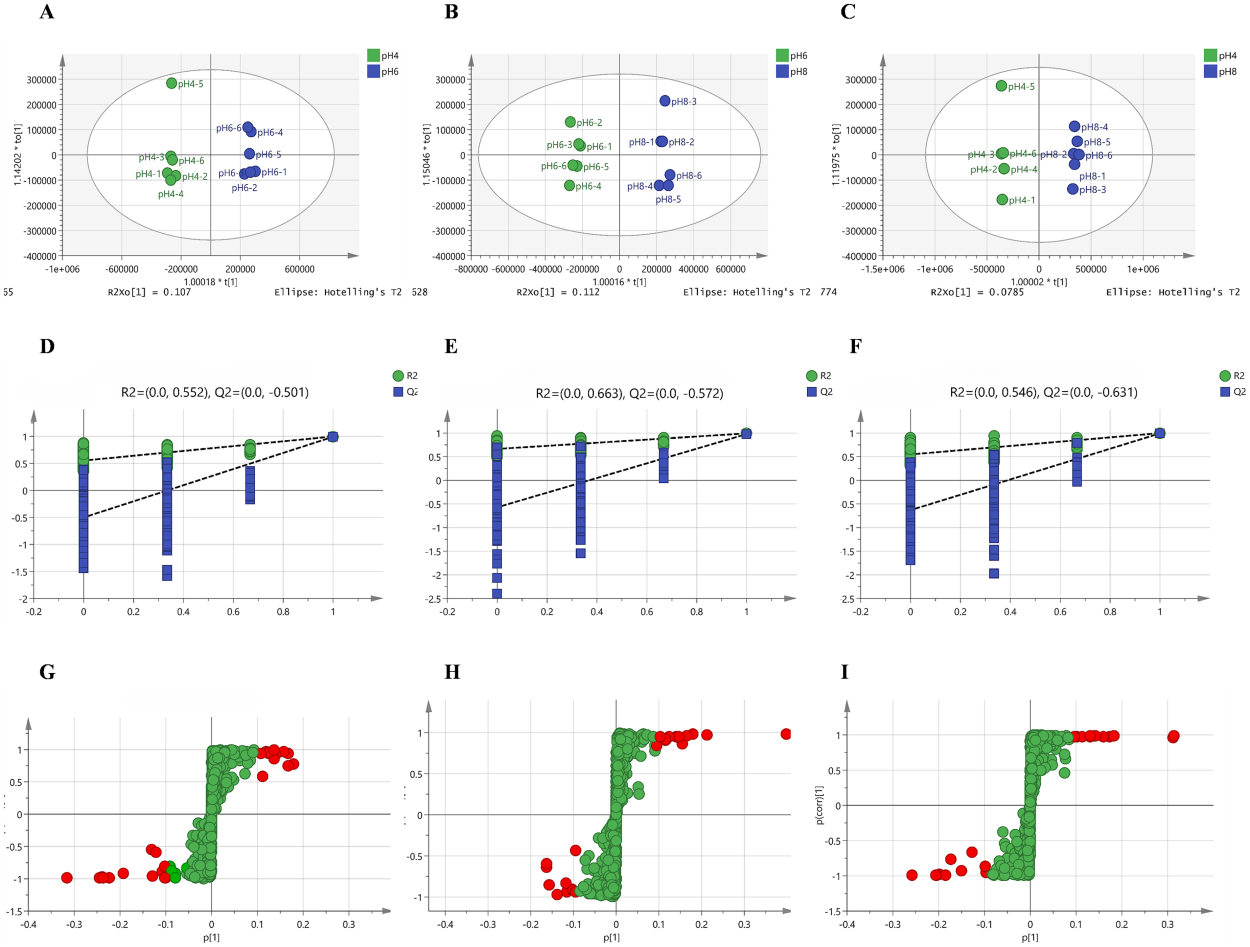
OPLS-DA of samples obtained at pH 4.0, pH 6.0 and pH 8.0. The OPLS-DA model score scatter plots shown in **(A–C)** represent pH 4.0 vs. pH 6.0, pH 6.0 vs. pH 8.0 and pH 4.0 vs. pH 8.0, respectively. The OPLS-DA model replacement test diagrams presented in **(D–F)** corresponded to pH 4.0 vs. pH 6.0, pH 6.0 vs. pH 8.0 and pH 4.0 vs. pH 8.0, respectively. **(G–I)** are the S plots of the OPLS-DA model for pH 4.0 vs. pH 6.0, pH 6.0 vs. pH 8.0 and pH 4.0 vs. pH 8.0, respectively. Variables with VIP > 3 are highlighted with red dots.

*A. cristatus* exhibited distinct morphologies, reproductive modes and growth potential at different pH ranges. In order to explore the correlation between these features and fungal metabolites, non-target metabolome technology was performed. In this case, a total of 1,023 substances were identified from the mycelium samples of *A. cristatus* cultured at pH 4.0, 6.0 and 8.0 (Supplementary Table S1). Subsequent principal component analysis (PCA) showed clear separation of samples obtained at the different pH, with close clustering of their replicates. The two principal components, namely PC1 and PC2, could explain 65.1, 54.7 and 69.0% of the differences between pH 4.0 vs. pH 6.0, pH 6.0 vs. pH 8.0 and pH 4.0 vs. pH 8.0, respectively (Figure 3). Supervised orthogonal partial least squares discriminant analysis (OPLS-DA) further confirmed the above results, with clear separation of samples of different pH and close clustering of their six replicates (Figures 4A–C). In this case, R2Y and Q2 were both >0.9 (close to 1) (Supplementary Table S2), thus indicating that the model was highly interpretable and predictable. Moreover, the results of permutation tests showed that the y-intercepts of the Q2 regression line were all <0, thereby confirming the reliability of the model (Figures 4D–F). Finally, the two sets of variables in the S-plot always appeared in the upper right and lower left quadrants of the S-plot, with variables far from the center of the graph contributing more to the model classification (Figures 4G–I). Overall, the results of PCA and OPLS-DA analyses confirmed that the metabolic profile of *A. cristatus* varied depending on whether the fungus grew in acidic (pH 4.0), weakly acidic (pH 6.0) or weakly alkaline (pH 8.0) environments, thus suggesting that the biosynthesis of metabolites in *A. cristatus* was significantly influenced by the initial pH of the growth environment.

### 3.3 Differential metabolic substances of *Aspergillus cristatus* at different initial pH

**Figure 5:**
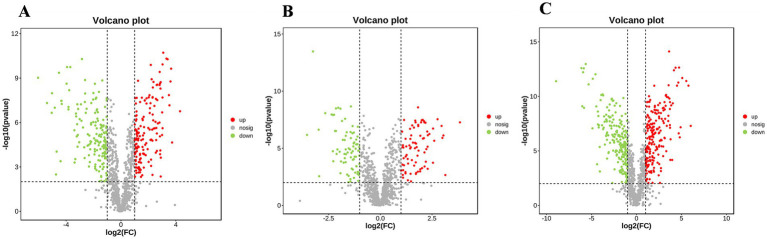
Volcano diagram analysis of samples obtained at pH 4.0, 6.0 and 8.0. **(A–C)** respectively represent volcano plot of differential substances of pH 4.0 vs. pH 6.0, pH 6.0 vs. pH 8.0 and pH 4.0 vs. pH 8.0.

**Figure 6:**
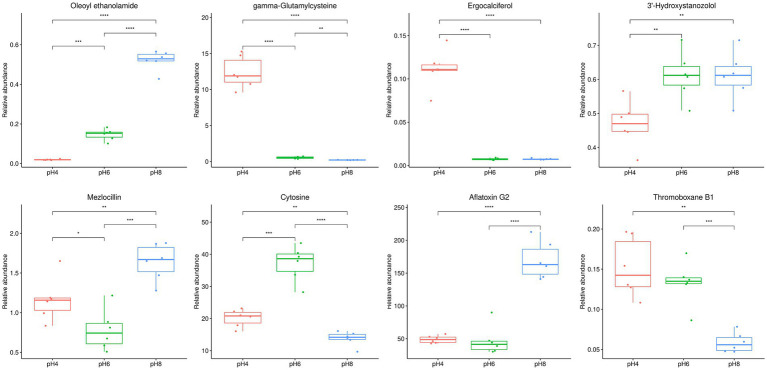
Eight types of box plot patterns reflecting the changes in *A. cristatus*’s metabolites. The name of the compound at figure just an example of many compounds represented. * Means *p* < 0.05, ** means *p* < 0.01, *** means *p* < 0.001, ****means *p* < 0.0001.

The 1,023 metabolites identified from the mycelial samples were screened based on the OPLS-DA analyses (Figures 4G–I) and volcano diagrams (Figure 5) (FC > 2 or FC < 0.5 and *p* < 0.05). In this case, pH 4.0 vs. pH 6.0, pH 6.0 vs. pH 8.0, and pH 4.0 vs. pH 8.0 comparisons resulted in the identification of 318, 171 and 405 significantly different metabolites, respectively (Supplementary Tables S3–S5). Additionally, pH 4.0 vs. pH 6.0, pH 6.0 vs. pH 8.0, and pH 4.0 vs. pH 8.0 presented 57 common differential substances and had 42, 34 and 85 unique differential substances, respectively (Supplementary Figure S3). Overall, the results showed that, as the pH changed from 4.0 to 6.0 to 8.0, eight types of changes were recognized in the pattern of *A. cristatus*’s metabolites. Firstly, some compounds, such as oleoyl ethanolamide, were continuously up-regulated, while others, such as *γ*-glutamylcysteine, were continuously down-regulated. Similarly, certain metabolites were either down-regulated (e.g., ergocalciferol) or up-regulated (e.g., 3′-hydroxystanozolol), but remained at a constant level in pH6 and pH8, while others were up-regulated in pH4 to pH6 and down-regulated (e.g., mezlocillin) in pH6 to pH8 or vice-versa (e.g., cytosine). Finally, a group of metabolites remained at unchanged levels in pH4 to pH6, but up-regulated (e.g., aflatoxin G2) or down-regulated (e.g., thromoboxaneB1) in pH6 to pH8 (Figure 6). These significantly different substances could also be assigned to eight specific categories, namely amino acids and their derivatives, nucleosides, flavonoids and flavonoids, vitamins, fatty acids and their derivatives, organic acid compounds, carbohydrates and phospholipids.

### 3.4 Correlation analysis between differential substances and vegetative growth

**Figure 7:**
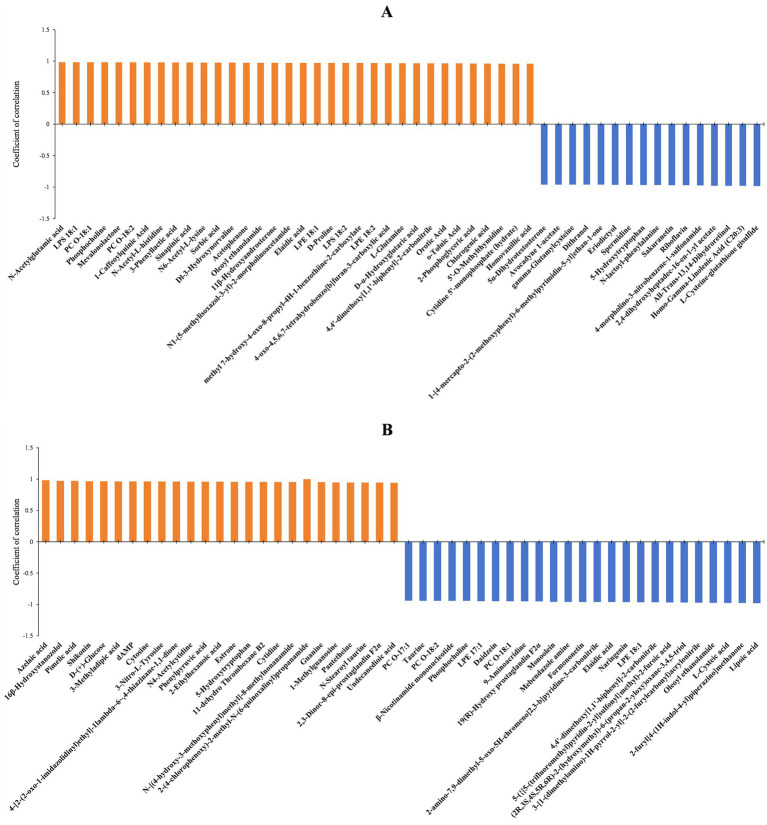
Correlation analysis between the identified differential substances and biomass at pH 4.0 vs. pH 6.0 **(A)** and pH 6.0 vs. pH 8.0 **(B)**.

**Figure 8:**
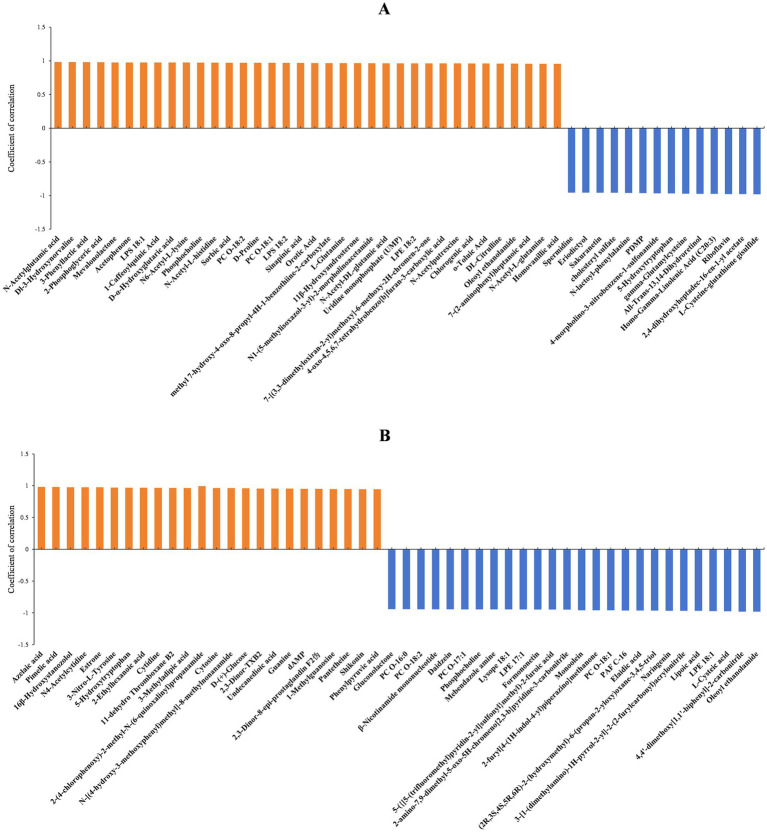
Correlation analysis between differential substances identified at pH 4.0 vs. pH 6.0 **(A)** and pH 6.0 vs. pH 8.0 **(B)** and colony diameter.

The diameters of fungal colonies reflect the growth rate of mycelia under specific environmental conditions. Similarly, the biomass can indicate the vegetative growth of substrate and aerial hyphae under those conditions. In this study, *A. cristatus* was cultured under similar conditions except for pH, and differences in colony diameter and biomass were noted, indicating that environmental pH not only affected the radial growth of *A. cristatus*, but also influenced the vegetative growth of aerial hyphae. Through correlation analysis, the top 50 substances that could be linked to the radial growth and biomass of *A. cristatus* were screened. In pH 4.0 vs. pH 6.0, 34 differential substances that were positively correlated with colony biomass (Figure 7A), while 36 differential substances were positively correlated with colony diameter (Figure 8A). Of these, 29 substances including N-acetylglutamic acid, LPS (18:1), PC (18:1), phosphocholine and 1-caffeoylquinic acid were common to both morphological characteristics, while twelve compounds, including N-acetyl-DL-glutamic acid, DL-citrulline and N-acetyl-L-glutamine were different. Furthermore, 16 differential substances were negatively correlated with colony biomass, and 14 exhibited a negative correlation with colony diameter. In this case, 12 substances, including spermidine, eriodictyol, sakuranetin, gamma-glutamylcysteine and L-cysteine-glutathione gisulfide were similar between the two, while 6 substances, including cholesteryl sulfate and 5α-dihydrotestosterone were different. In addition, pH 6.0 vs. pH 8.0 identified 25 differential substances that were positively correlated with colony biomass (Figure 7B) as well as 24 differential substances that were positively correlated with colony diameter (Figure 8B). In particular, 23 substances, such as shikonin, shenylpyruvic acid and 2’-Deoxyadenosine-5′-monophosphate (dAMP), were common to both groups, while 3 compounds, such as 2,3-dinor-thromboxane B2 (2,3-dinor-TXB2) and N-stearoyl taurine, were different. There were also 25 differential substances that were negatively correlated with colony biomass, with an additional 26 differential substances exhibiting a negative correlation with colony diameter. In this case, 22 substances, including LPE (18:1), oleoyl ethanolamide, L-cysteic acid, monoolein, daidzein, mebendazole amine, formononetinand and naringenin were similar to both groups, while 7 substances, such as gluconolactone, PC (16:0) and taurine were different. These results showed that most of the differential substances associated with the vegetative growth of substrate and aerial hyphae (including positive and negative correlations) were similar in both pH 4.0 vs. pH 6.0 and pH 6.0 vs. pH 8.0. It is suggested that the material basis for vegetative growth of substrate and aerial hyphae of *A. cristatus* is almost the same.

## 4 Discussion

### 4.1 *Aspergillus cristatus’*s strategy to adapt to pH environments that are lower than the optimal one

Under such conditions, the cells typically consume energy through proton pumps located on the plasma membrane to expel H^+^ and maintain pH_i_ homeostasis (Guan and Liu, 2020). For *A. cristatus*, pH 4.0 represents a stressful environment where the amount of H^+^ largely exceeds the balance necessary for its normal growth. In this study, it was found that, at pH 4.0, *A. cristatus* accumulated several substances that were related to energy metabolism –adenosine (Fredholm et al., 2011; Laborit and Bonifacj, 1984), adenosine succinate (Arinze, 2005), riboflavin and riboflavin-5-phosphate (Powers et al., 2012) – compared with pH 6.0. Regarding the differential metabolic pathways, adenosine was primarily enriched in nucleotide metabolism and purine metabolism, while adenylocuccinic acid was enriched in purine metabolism, nucleotide metabolism as well as the biosynthesis of cofactors. Similarly, riboflavin and riboflavin-5-phosphate being mainly enriched in the biosynthesis of cofactors and the Riboflavin metabolism (Supplementary Table S6). These substances promote energy synthesis when the cell energy is low (Arinze, 2005; Laborit and Bonifacj, 1984; Fredholm et al., 2011; Powers et al., 2012). Furthermore, the vegetative growth of *A. cristatus* was significantly lower at pH 4.0 than that at pH 6.0. Similar to our results, Zheng et al. (2011) reported that the biomass formation of *Saccharomyces cerevisiae* cells under acetic acid stress was less than that in the unstressed condition. To counteract this stress, *S. cerevisiae* cells expelled proton by increasing H-ATPase activity, resulting in a significant loss of available energy for growth and other essential metabolic functions. Therefore, it is speculated that *A. cristatus* used most of energy for expelling H^+^ to reduce its intracellular concentration, resulting in impaired available energy for cell proliferation and growth at pH 4.0, while the accumulation of adenosine and other substances is a substance strategy to improve energy metabolism to maintain survival.

Additionally, in response to the stress of pH 4.0, *A. cristatus* had to modify the metabolic pathways and fluxes of some substances to repair and mitigate damage. In this context, one of the most significant changes was the increase in the synthesis of reduced glutathione (GSH) which was 42 times and 490 times higher than at pH 6.0 and 8.0, respectively, and the direct precursor of glutathione, namely gamma-glutamylcysteine, also accumulated at pH 4.0 (Verified, Supplementary Figure S4). GSH was mainly enriched in three differential metabolic pathways: glutathione metabolism, cysteine and methionine metabolism as well as the biosynthesis of cofactors, while gamma-glutamylcysteine was enriched in glutathione metabolism and the biosynthesis of cofactors pathways (Supplementary Table S6). As a major endogenous biological antioxidant, GSH can achieve antioxidant defense in cells by directly interacting with ROS and providing electrons to other redox systems to repair oxidative damage to cells (Pócsi et al., 2004; Even et al., 2003; Meyer, 2008). On the other hand, gamma-glutamylcysteine can effectively detoxify hydrogen peroxide and superoxide anion (Quintana-Cabrera et al., 2012). Therefore, it is speculated that *A. cristatus* experiences a stronger oxidative stress response at pH 4.0 than at pH 6.0, as well as the excessive synthesis of GSH and its direct precursor is likely a defense mechanism to counteract oxidative stress caused by the high H^+^ concentrations in an acidic environment. Similarly, the protective effect of GSH on cells under acid stress has also been reported in other microorganisms. For example, Zhang et al. (2007) showed that GSH can protect *Lactococcus lactis* against a long-term mild acid challenge (pH 4.0) and a short-term severe acid challenge (pH 2.5). As well as, Wang et al. (2015) found that the accumulation of glutathione in *Candida utilis* can better maintain the homeostasis of intracellular pH under acid stress. Furthermore, at pH 4.0, the excess H^+^ in the environment may disrupt the cell’s normal redox potential, affecting the electron transport chain and respiration, thereby threatening survival. GSH, which is mainly found in the nucleus, mitochondria and endoplasmic reticulum, forms distinct redox pools that can independently regulate redox potential and cellular activity (Marí et al., 2009; Aoyama and Nakaki, 2015). Therefore, it is speculated that the accumulation of GSH in *A. cristatus* at pH 4.0 helps to balance the redox potential of organelles, such as mitochondria, and maintain intracellular homeostasis. Under low pH stress, *A. cristatus* accumulates not only large amounts of GSH but also other antioxidant compounds, such as flavonoids (Stompor, 2020; Islam et al., 2020; Singh et al., 2021) (e.g., sakuranetin, eriodictyol and puerarin) and vitamins (Blaner et al., 2021; Dal-Pizzol et al., 2001) (e.g., vitamin A, all-trans-13,14-dihydroretinol and L-ascorbate). These suggest that *A. cristatus* can activate a broad antioxidant defense system involving various different compounds, with each potentially contributing to distinct antioxidant mechanisms.

When fungi encounter environmental stress, they often produce various compatible solutes, such as amino acids, soluble carbohydrates, polyols and other substances, to protect cells from damage (Danilova et al., 2020; Bondarenko et al., 2017; Liao et al., 2013). These compatible solutes help fungi to adapt to challenging environment while maintaining their capacity for growth and reproduction. For example, *Streptomyces roseosporus* (Liao et al., 2013) increases trehalose under quinic acid stress, with the alkaliphilic fungus *S. tronii* (Bondarenko et al., 2017) also accumulating large amounts of this compound under acidic conditions. Similarly, *Emericellopsis alkalina* (Danilova et al., 2020) can accumulate mannitol at a pH of 4.5. In this study, at pH 4.0, *A. cristatus* synthesized and accumulated large amounts of soluble carbohydrates, such as raffinose (Salvi et al., 2022), trehalose, lactose and maltose. In particular, their levels increased by dozens of times under the acidic conditions, hence suggesting that these substances were among the most important compatible solutes which enabled *A. cristatus* to tolerate the low pH stress.

### 4.2 *Aspergillus cristatus*’s strategy to cope with pH environments that are higher than the optimal one

This study showed that, at pH 8.0, *A. cristatus* adopted a different growth pattern for adapting to a high pH stress environment. In this case, the fungus not only accumulated large amounts of phosphatidylcholine (PC), including PC (16: 1), PC (17:1), PC (18:2) and PC (18:1), but also synthesized large numbers of unsaturated lysophospholipids (LPLs), such as LPE (17:1), LPE (16:1), LPE (18:1), LPC (17:2), LPC (16:1), LPS (18:2), LPS (20:4) and other lipid molecules alongside monoolein, oleic acid and oleoyl ethanolamide. Of these, PC, which constitutes eukaryotic biofilms, represents one of the main phospholipids, accounting for about 50% of membrane lipids (de Kroon, 2007). It also significantly influences membrane integrity and fluidity, while those containing unsaturated fatty acid acyl chains are more likely to enhance membrane permeability (McMaster, 2018). In addition, LPLs, a secondary membrane component and signal medium (Drzazga et al., 2014), while monoolein and oleic acid are high lipophilic skin penetration enhancers that increase lipid fluidity (Ruela et al., 2016). In an alkaline environment, the lack of protons may lead to an imbalance in the membrane potential, thus affecting membrane permeability and material transport (Kane, 2016). Bondarenko et al. (2017) reported that the alkaliphilic fungus *S. tronii* may accumulate phosphatidic acid in membrane lipids at a pH of 10.2. Therefore, it is likely that the accumulation of these substances under the alkaline conditions could be involved in repairing the cell membrane, improving its permeability and fluidity, maintaining the normal transport of nutrients and maintaining the active physiological state of the cell membrane.

*A. cristatus* also accumulated significant acidic substances, such as 3-methylglutaric acid, D-gluconic acid and quinic acid, at pH 8.0, and these are expected to assist the fungus in coping with the environmental pH. It has been reported that *A. carbonarius* (Barda et al., 2020), where gluconic acid and citric acid were produced under alkaline conditions to reduce the impact of environmental pH. In addition, *A. cristatus* also had oxidative stress repair at pH 8, and the oxidative stress damage was reduced by accumulating antioxidant substances, such as formononetin, daidzein and naringenin (Tang et al., 2020; Gao et al., 2021). Obviously, these substances are different from the accumulation of glutathione and other substances at pH4, which suggests that although both low pH stress and high pH stress activate the anti-oxidative stress system, the substances involved are different and the oxidative defense system may also be different.

## 5 Conclusion

When *A. cristatus* grows under unsuitable low or high pH conditions, its vegetative growth, asexual sporulation and biosynthesis metabolism undergo significant changes, and this is reflected in the diverse changes that occur at the metabolic level. Moreover, the substance basis for the vegetative growth of the substrate hyphae and aerial hyphae of the bacteria is similar. In addition, *A. cristatus* adopts different adaptation strategies to cope with low pH stress and high pH stress. Under low pH stress, cells encounter strong oxidative stress damage, which is mainly dealt with by three different strategies, namely sacrificing the energy required for vegetative growth to maintain the balance of pH_i_, activating glutathione antioxidant defense system, and secreting compatible solutes. On the other hand, under high pH stress, cells undergo cell membrane damage alongside changes in membrane permeability. In this case, *A. cristatus* adopts a different three-dimensional substance response strategy, which involves synthesizing a large number of lipid molecules, such as PCs, that contain unsaturated fatty acid acyl chains, LPLs and oleic acid. It also synthesizes acidic substances to reduce environmental pH while initiating an antioxidant defense system composed of formononetin and other substances.

## Data Availability

The original contributions presented in the study are included in the article/Supplementary material, further inquiries can be directed to the corresponding author.

## Footnotes

^1^ https://www.mzcloud.org/

^2^ https://www.genome.jp/kegg/pathway.html

^3^ https://hmdb.ca/metabolites

^4^ http://www.lipidmaps.org/

^5^ https://www.omicshare.com/
