# Supplementary material for: Metabolite profiling and adaptation mechanisms of Aspergillus cristatus under pH stress
Source: Front Microbiol. 2025 Apr 1;16:1576132. doi: 10.3389/fmicb.2025.1576132 (PMC11998282; doi:10.3389/fmicb.2025.1576132)
Supplement: Supplementary file 2 [file Data_Sheet_1.DOCX]

**Supplementary Figure 1.** Morphological characteristics of *Aspergillus cristatus* at pH 3.0-8.0. **a-f**: Front of the colony at pH 3.0-8.0, **g-l**: back of the colony at pH 3.0-8.0.

**Supplementary Figure 2.** The colony diameter of *A. cristatus* after 12 days of growth on media of pH 3.0-8.0.

**Supplementary Figure 3.** Venn diagram showing the differences between the samples of obtained at pH 4.0, 6.0 and 8,0.

**1.Gamma-glutamylcysteine synthetase enzyme activity validation**

After multivariate statistical analysis such as PCA and OPLS-DA, we found that the cumulative content of glutathione (GSH) and γ-glutamylcysteine (γ-GC) decreased significantly with the increase of pH from 4 to 6 and then to 8. After that, we detected the activity of γ-glutamylcysteine synthetase (γ-GCS) , the rate-limiting enzyme catalyzing the condensation of glutamate and cysteine to form γ-GC in GSH biosynthesis, found that the activity of γ-GCS decreased significantly with the increase of pH from 4 to 6 and then to 8 (Supplementary Figure 4), which was consistent with the cumulative variation of GSH and γ-GC. The targeted metabolite validation is identified by enzyme activity analysis.

**Supplementary Figure 4**. Activity of γ-glutamylcysteine synthetase (γ-GCS) in *A. cristatus* at different pH values. ****means p ＜0.0001

**2.Quality assessment (QC) and quality control (QA)**

In the process of data quality evaluation, the distribution of PC1 values at all sample points can be used to evaluate whether the laboratory sample preparation and sample measurement processes are in a controllable state. Sample points exceeding the control limit (3 times standard deviation) are considered outliers. As shown in **Supplementary Figure 5**, generally speaking, all points will be within the control boundary. Among them, most points will fluctuate up and down around the mean axis within 2 times the standard deviation, and a small number of points will approach the control limit.

Quality control is one of the fundamental concepts in biological analysis, used to ensure the repeatability and accuracy of omics data. Due to the direct contact between the chromatographic system and mass spectrometry with the sample, as the number of analyzed samples increases, the chromatographic column and mass spectrometry will gradually become contaminated, leading to signal drift and causing systematic measurement errors. The behavior of tracking the entire data collection process by repeatedly using the same quality control sample (QC sample) has been recommended and used by most experts in the field of analytical chemistry. Quality control samples are used to evaluate the signal drift of the entire mass spectrometry data during the collection process, which can be further identified, corrected, and improved by precise algorithms to enhance the quality of the data. This process uses the QC-RFSC algorithm of the Stat Target package in R language to correct the signal peaks of each sample's features (each metabolite), and records the correction effect of each metabolite. QC samples are samples obtained by mixing equal amounts of all samples. During the signal data collection process, QC samples are inserted at the beginning, end, and middle positions to record signal drift. All QC samples are the same. If there is no signal drift, the signal strength of QC samples should remain unchanged during the data acquisition process. As shown in **Supplementary Figure 6**, after correcting the signal drift, if the QC sample points gather together in the PCA plot, it proves that the correction effect is good.


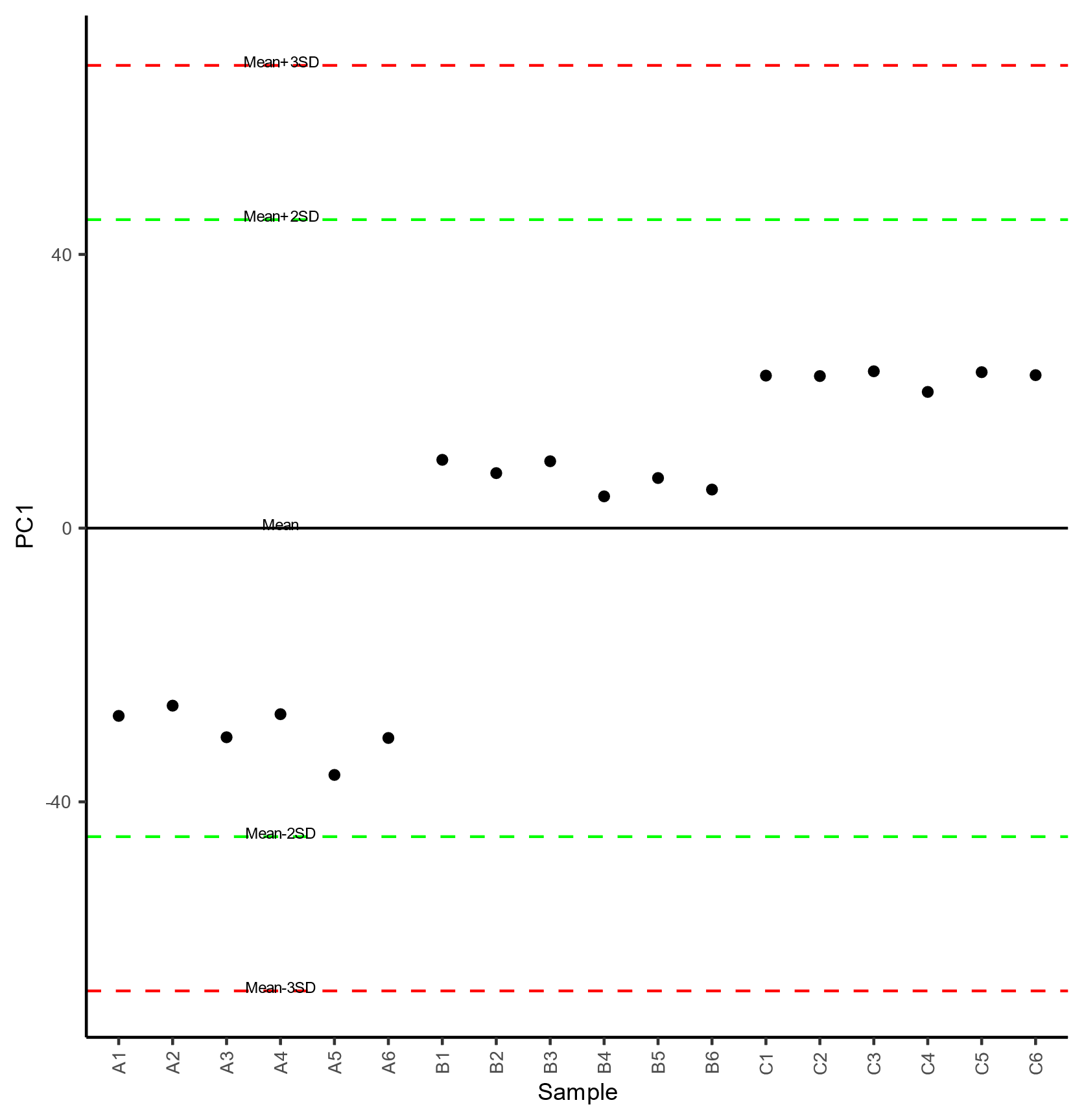


**Supplementary Figure 5.** All Sample_PC1. A, B and C represent pH4, pH6 and pH8, respectively.

**Supplementary Figure 6.**  neg _PCA_QC (**A**); pos _PCA_QC (**B**). A, B and C represent pH 4.0, pH 6.0 and pH 8.0, respectively.

**3.Normalization**

Within sample correction: dividing the abundance of all features in the sample by the median abundance of the sample (similar to relative abundance calculation); This is to correct the library size. During the measurement process, there is a significant difference in the total metabolite content obtained from each sample, which is usually a systematic error caused by the sample collection and measurement process. Dividing by the median, mean, or total is a common way to correct this systematic error.

Content matrix correction: log conversion is performed on all content values; T test and ANOVA methods for comparing differences require that metabolite content follows a normal distribution. Therefore, we generally use log transformation to make the distribution of metabolite content close to a normal distribution.

Feature internal correction: subtracting the mean abundance of all samples corresponding to the feature and then dividing it by the standard deviation of the feature abundance; The purpose of feature internal correction is to ensure that the mean and standard deviation (or median, quartile, scale) of all metabolites are at the same level; Analysis such as PCA, PLSDA, OPLSDA, and machine learning, if metabolite standardization is not carried out, the importance of metabolites with high mean and standard deviation will tend to be higher than that of metabolites with low mean and standard deviation. Such results are obviously not what we want, and only those with large differences between groups should have high importance. As shown in **Supplementary Figure 7**, before standardization correction, the median and upper and lower quartiles of metabolite content varied greatly, but after standardization correction, they were basically at the same level.


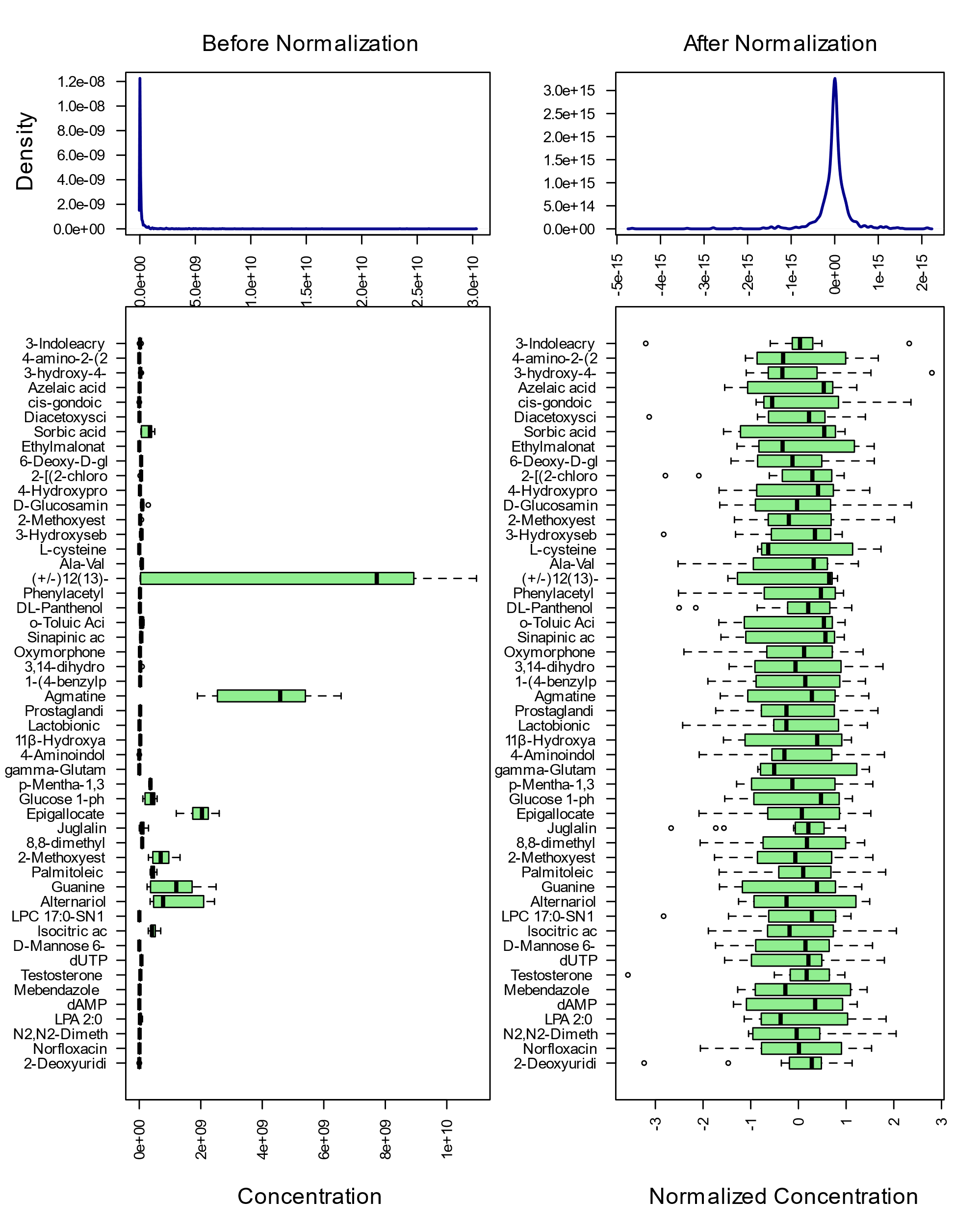


**Supplementary Figure 7.** Compound wise normalization.

**4.Significantly different substances could be assigned to eight specific categories**

**Amino Acids and Their Derivatives**

pH 4.0 vs pH 6.0 had 44 amino acids and their derivatives. Compared with pH 4.0, 33 substances were up-regulated, and 11 substances were down-regulated in pH 6.0. Among them, the contents of levodopa, DL-3-hydroxynorvaline, *N*-phenylacetylglutamine and *N*-acetyl-DL-phenylalanine were up-regulated by 11.31, 9.995, 8.035 and 7.374 times, respectively, whereas the contents of L-glutathione (GSH), gamma-glutamylcysteine, cysteinylglycine and L-cysteine were down-regulated by 42.004, 22.959, 8.082 and 5.038 times, respectively.

pH 6.0 vs pH 8.0 had 8 amino acids and their derivatives. Compared with pH 6.0, three substances were up-regulated, and five substances were down-regulated in pH 8.0. Among them, the contents of L-cysteic acid, *N*-acetyl-DL-phenylalanine and taurine were up-regulated by 5.184, 4.055 and 2.165 times, respectively, whereas the contents of GSH, L-methionine methyl seter, 5-hydroxytryptophan and gamma-glutamylcysteine were down-regulated by 11.667, 5.377, 2.242 and 2.525 times.

pH 4.0 vs pH 8.0 had 58 amino acids and their derivatives. Compared with pH 4.0, 43 substances were up-regulated, and 15 substances were down-regulated in pH 8.0. Among them, the contents of *N*-acetyl-DL-phenylalanine, L-cysteic acid, *N*-acetylneuraminic acid, Dl-3-hydroxynorvaline and taurine were up-regulated by 29.903, 25.151, 13.821, 12.655 and 9.920 times, respectively, whereas the contents of GSH, gamma-glutamylcysteine, cysteinylglycine and L-cysteine were down-regulated by 490.059, 57.976, 8.763 and 8.463 times, respectively.

Among them, the contents of *N*-acetyl-DL-phenylalanine, L-cysteic acid and taurine were continuously up-regulated with the increase of pH. The contents of GSH, gamma-glutamylcysteine and 5-hydroxytryptophan were continuously down-regulated with the increase of pH.

**Nucleosides**

pH 4.0 vs pH 6.0 had 21 nucleoside differential substances. Compared with pH 4.0, pH 6.0 had ten up-regulated and eleven down-regulated substance. Among them, cytidine 5'-monophosphate, adenosine 5'-monophosphate and 5'-O-methylthymidine were up-regulated by 7.013, 5.116 and 2.976 times, respectively, whereas 2'-O-methyladenosine, adenosine, adenylocuccinic acid and XMP (5'-Xanthylic acid) were down-regulated by 33.095, 14.217, 8.972 and 2.239 times, respectively.

pH 6.0 vs pH 8.0 had 10 nucleoside differential substances. Compared with pH 6.0, pH 8.0 had four up-regulated and six down-regulated substances. Among them, β-nicotinamide mononucleotide and adenylocuccinic acid were up-regulated by 3.293 and 2.563 folds, respectively, whereas 2'-deoxyadenosine-5'-monophosphate (dAMP), cytidine, 1-methylguanosine and XMP were down-regulated by 3.806, 3.294, 2.239 and 3.517 times, respectively.

pH 4.0 vs pH 8.0 had 32 nucleoside differential substances. Compared with pH 4.0, pH 8.0 had 13 up-regulated and 19 down-regulated substances. Among them, cytidine 5'-monophosphate, adenosine 5'-monophosphate and β-nicotinamide mononucleotide were up-regulated by 13.131, 7.796 and 4.630 folds, respectively, whereas 2'-O-methyladenosine, adenosine and XMP were down-regulated by 56.989, 13.054 and 7.875 times, respectively.

Among them, the contents of 1-methylguanosine and XMP continued to decrease with the increase of pH. No nucleosides continued to increase with the increase of pH.

**Flavonoids and Isoflavonoids**

pH 4.0 vs pH 6.0 had 13 flavonoids as differential substances. Compared with pH 4, pH 6.0 had two up-regulated and 13 down-regulated substances. Among which catechin and formononetin were up-regulated by 7.176 and 4.929 times. Sakuranetin, eriodictyol and Puerarin were down-regulated by 14.164, 10.529 and 4.532 times, respectively.

pH 6.0 vs pH 8.0 had 7 flavonoids as differential substances. Compared with pH 6.0, pH 8.0 had five up-regulated and two down-regulated substances. Among which formononetin and daidzein were up-regulated by 3.188 and 2.710 times, respectively. Sakuranetin and eriodictyol were down-regulated 4.910 and 3.804 times, respectively.

pH 4.0 vs pH 8.0 had 15 flavonoids as differential substances. Compared with pH 4.0, pH 8.0 had 5 up-regulated and 10 down-regulated substances. Among which formononetin and catechin were up-regulated by 15.711 and 6.843 times, respectively. Sakuranetin, eriodictyol and puerarin were down-regulated by 69.545, 40.049 and 7.904 times, respectively.

Among them, the content of formononetin continued to increase with the increase of pH, whereas the contents of sakuranetin and eriodictyol decreased continuously with the increase of pH.

**Vitamins**

pH 4.0 vs pH 6.0 had 11 different vitamins. Compared with pH 4.0, pH 6.0 had three up-regulated and eight down-regulated substances. Among them, biotin, vitamin E acetate and pyridoxine were up-regulated by 3.062, 2.473 and 2.147 times, respectively, whereas ergocalciferol, all-trans-13,14-dihydroretinol and vitamin A were down-regulated by 15.213, 7.174 and 4.726 times, respectively.

pH 6.0 vs pH 8.0 had 3 different vitamins. Compared with pH 6.0, pH 8.0 had two up-regulated and one down-regulated substances. Among them, flavin adenine dinucleotide and lipoic acid were up-regulated by 2.537 and 3.541 times, respectively, whereas vitamin E acetate was down-regulated by 2.076 times.

pH 4.0 vs pH 8.0 had 12 different vitamins. Compared with pH 4.0, pH 8.0 had five up-regulated and seven down-regulated substances. Among them, biotin, flavin adenine dinucleotide and pyridoxine were up-regulated by 5.646, 2.776 and 2.724 times, respectively, whereas ergocalciferol, all-trans-13,14-dihydroretinol and vitamin A were down-regulated by 15.068, 6.823 and 6.021 times, respectively.

Vitamin differential substances were not continuously up-regulated or down-regulated with the increase of pH.

**Fatty acid and Derivatives**

pH 4.0 vs pH 6.0 included 13 fatty acids and their derivatives. Compared with pH 4.0, seven substances were up-regulated, and six substances were down-regulated in pH 6.0. Among them, the contents of 7- (2-aminophenyl) heptanoic acid, oleoyl ethanolamide and elaidic acid were up-regulated by 11.613, 7.458 and 5.183 times, whereas the contents of homo-gamma-Linolenic acid (C20:3), *N*-steroyl taurine and cis-gondoic acid were down-regulated by 7.174, 4.071 and 3.242 folds, respectively.

pH 6.0 vs pH 8.0 included 22 fatty acids and their derivatives. Compared with pH 6.0, six substances were up-regulated, and 16 substances were down-regulated in pH 8.0. Among them, the contents of oleic acid, elaidic acid and oleoyl ethanolamide were up-regulated by 4.301, 3.918 and 3.560 times, whereas the contents of 3-methyladipic acid and 2-ethylhexanoic acid were down-regulated by 7.905 and 6.327 times, respectively.

pH 4.0 vs pH 8.0 included 28 fatty acids and their derivatives. Compared with pH 4.0, ten substances were up-regulated, and 18 substances were down-regulated in pH 8.0. Among them, the contents of oleoyl ethanolamide, elaidic acid and 7-(2-aminophenyl) heptanoic acid were up-regulated by 26.549, 20.307 and 6.614 times, whereas the contents of 3-methyladipic acid, *N*-stearoyl taurine and homo-gamma-linolenic acid (C20:3) were down-regulated by 8.654, 8.535 and 6.823 folds, respectively.

Among them, the contents of oleoyl ethanolamide and elaidic acid continued to increase with the increase of pH. No fatty acids and their derivatives decreased continuously with increasing pH.

**Organic acids**

pH 4.0 vs pH 6.0 had 33 organic acid compounds. Compared with pH 4.0, 24 substances were up-regulated, and 11 substances were down-regulated in pH 6.0. Chlorogenic acid, mevalonolactone, o-toluic acid and 3-methylglutaric acid were up-regulated by 8.502, 7.043, 6.752 and 2.410 folds. In contrast, D- (-) -quinic acid, kynurenic acid and phenylpyruvic acid were down-regulated by 8.826, 3.817 and 3.590 times, respectively.

pH 6.0 vs pH 8.0 had 11 organic acid compounds. Compared with pH 6.0, five substances were up-regulated, and six substances were down-regulated in pH 8.0. 3-methylglutaric acid, D-gluconic acid and D-(-)-quinic acid were up-regulated by 2.504, 2.340 and 2.322 times, respectively. In contrast, phenylpyruvic acid, 4-acetamidobutyric acid and 2-isopropylmalate were down-regulated by 2.769, 2.636 and 2.516 times, respectively.

pH 4.0 vs pH 8.0 had 48 organic acid compounds. Compared with pH 4.0, 33 substances were up-regulated and 15 substances were down-regulated in pH 8.0. Chlorogenic acid, mevalonolactone, gentisic acid and o-toluic acid were up-regulated by 10.373, 8.824, 8.202 and 7.997 times, respectively. In contrast, phenylpyruvic acid, kynurenic acid and 2-isopropylmalate were down-regulated by 9.940, 5.647 and 3.973 times, respectively.

Among them, the content of 3-methylglutaric acid was constantly increased with the increase of pH, while the content of phenylpyruvic acid continued to decrease with the increase of pH.

**Carbohydrate**

pH 4.0 vs pH 6.0 had 16 carbohydrates. Compared with pH 4.0, pH 6.0 had five up-regulated and 11 down-regulated substances. Among them, the contents of 3-phosphoglyceric acid, 2-phosphoglyceric acid and 2-deoxyglucose-6-phosphate were up-regulated by 4.594, 3.594 and 2.713 times, respectively, whereas the contents of D-raffinose, α, α-trehalose, D- (+) -maltose, α-Lactose, maltotetraose, maltotriose and D- (+) -glucose were down-regulated by 65.836, 26.663, 25.816, 21.160, 20.180, 19.947 and 4.941 times, respectively.

pH 6.0 vs pH 8.0 had four carbohydrates. Compared with pH 6.0, pH 8.0 had three up-regulated and one down-regulated substances. Among them, the content of 6-sialyllactose was up-regulated by 4.308 folds, whereas the content of D- (+) -glucose was down-regulated by 2.534 times.

pH 4.0 vs pH 8.0 had 16 carbohydrates. Compared with pH 4.0, pH 8.0 had nine up-regulated and seven down-regulated substances. Among them, the contents of 3-phosphoglyceric acid and 2-phosphoglyceric acid were up-regulated by 6.562 and 4.933 times, respectively, whereas the contents of D-raffinose, maltotriose, maltotetraose, α, α-trehalose, α-Lactose and D- (+) -glucose were down-regulated by 65.209, 18.380, 18.109, 16.808, 16.800 and 12.520 times, respectively.

Among them, the content of D- (+) -glucose continued to decrease with the increase of pH, and no carbohydrates continued to increase with increasing pH.

**Phospholipid**

pH 4.0 vs pH 6.0 had 32 phospholipid differential substances. Compared with pH 4.0, pH 6.0 had 20 up-regulated and 12 down-regulated substances. Among them, the contents of phosphatidylcholine 18:2 [PC (18:2)], PC (18:1), lysophosphatidyl inositol 18:1 [LPI (18:1)], lysophosphatidyl serine 18:2[LPS (18:2)], lysophosphatidyl ethanolamine 18:1 [LPE (18:1 )] and lysophosphatidyl choline 18:1 [LPC (18:1)] were up-regulated by 10.579, 8.201, 5.823, 8.001, 5.020 and 2.090 times, respectively; whereas the contents of glycerophospho-*N*-palmitoyl ethanolamine and LPE (18:0) were down-regulated by 10.409 and 5.047 times, respectively.

pH 6.0 vs pH 8.0 had 32 phospholipid differential substances. Compared with pH 6.0, pH 8.0 had 31 up-regulated and one down-regulated substances. Among them, the contents of PC (18:1), PC (18:2), LPI (18:1), LPS (18:2), LPE (19:1) and LPC (18:1) were up-regulated by 4.146, 4.384, 4.820,3.222,3.569 and 2.722 times, respectively, whereas the content of LPC [(20:1)-SN1] was down-regulated by 2.099 times.

pH 4.0 vs pH 8.0 had 31 phospholipid differential substances. Compared with pH 4.0, pH 8.0 had 26 up-regulated and 5 down-regulated substances. Among them, the contents of PC (18:2), PC (18:1), LPI (18:1), LPS (18:1), LPE (18:1) and LPC (18:1) were up-regulated by 46.385, 34.000, 28.070, 25.804, 17.915 and 5.688 times, whereas the contents of lysophosphatidic acid 16:0 [LPA (16:0)], LPA (2:0) and LPC (12:1) were down-regulated by 9.762, 6.874 and 5.596 folds, respectively.

Among them, 19 phospholipids, such as PC (18:1), PC (18:2), LPI (18:1), LPS (18:2), LPE (18:1) and LPC (18:1), were continuously up-regulated with the increase of pH, and no phospholipids continued to decrease with the increase of pH.
